# Early Life Exposure to a Diet With a Supramolecular Lipid Structure Close to That of Mammalian Milk Improves Early Life Growth, Skeletal Development, and Later Life Neurocognitive Function in Individually and Socially Housed Male C57BL/6J Mice

**DOI:** 10.3389/fnins.2022.838711

**Published:** 2022-04-29

**Authors:** Steffen van Heijningen, Giorgio Karapetsas, Eline M. van der Beek, Gertjan van Dijk, Lidewij Schipper

**Affiliations:** ^1^Groningen Institute for Evolutionary Life Sciences, University of Groningen, Groningen, Netherlands; ^2^Department of Pediatrics, University Medical Center Groningen, University of Groningen, Groningen, Netherlands; ^3^Danone Nutricia Research, Utrecht, Netherlands

**Keywords:** early life nutrition, lipids, growth, skeletal development, cognition, behavior

## Abstract

Breastfeeding (duration) can be positively associated with infant growth outcomes as well as improved cognitive functions during childhood and later life stages. (Prolonged) exposure to optimal lipid quantity and quality, i.e., the supramolecular structure of lipids, in mammalian milk, may contribute to these beneficial effects through nutritional early-life programming. In this pre-clinical study, we exposed male C57BL/6J mice from post-natal Days 16 to 42 (i.e., directly following normal lactation), to a diet with large lipid droplets coated with bovine milk fat globule membrane-derived phospholipids, which mimic more closely the supramolecular structure of lipid droplets in mammalian milk. We investigated whether exposure to this diet could affect growth and brain development-related parameters. As these outcomes are also known to be affected by the post-weaning social environment in mice, we included both individually housed and pair-wise housed animals and studied whether effects of diet were modulated by the social environment. After Day 42, all the animals were fed standard semi-synthetic rodent diet. Growth and body composition were assessed, and the mice were subjected to various behavioral tests. Individual housing attenuated adolescent growth, reduced femur length, and increased body fat mass. Adult social interest was increased due to individual housing, while cognitive and behavioral alterations as a result of different housing conditions were modest. The diet increased adolescent growth and femur length, increased lean body mass, reduced adolescent anxiety, and improved adult cognitive performance. These effects of diet exposure were comparable between individually and socially housed mice. Hence, early life exposure to a diet with lipid droplets that mimic the supramolecular structure of those in mammalian milk may improve adolescent growth and alters brain function in both socially and individually housed mice. These findings suggest that lipid structure in infant milk formula may be a relevant target for nutritional solutions, targeting both healthy infants and infants facing growth challenges.

## Introduction

The first 1,000 days in the life of an infant is a period of fast growth and development, during which nutrition and other environmental factors can influence the growth rate and the structural and functional development of organs and tissues, with long-term consequences for their function. During at least the first 6 months after birth, the sole source of nutrition for an infant is human milk (HM) or infant milk formula (IMF) if HM is not available. It is well-known that an infant-feeding mode influences growth trajectories. Breastfed infants show more rapid body weight gain in the first 2–3 months after birth compared to formula-fed infants, while formula-fed infants gain weight faster at a later stage (Butte et al., 2000; Kramer et al., 2002). Breastfeeding (duration) may be positively associated with parameters indicative of bone health during infancy and adolescence (Andres et al., 2013; Jones et al., 2013; Muniz et al., 2015; Blanco et al., 2017). Furthermore, there are numerous publications showing that breastfeeding exclusivity and duration are positively associated with neurodevelopmental outcomes, including improved brain structural development and myelination (Deoni et al., 2018; Kar et al., 2021), cognitive function, and school performance (Belfort et al., 2013; Horta et al., 2015; Leventakou et al., 2015; Hou et al., 2021; Lopez et al., 2021). Breastfeeding (duration) has been reported to be protective against mental health issues and behavioral/affective problems, including anxiety, psychosocial- and mental disorders throughout childhood and early adulthood (Hayatbakhsh et al., 2012; Huang et al., 2019), and is proposed to increase the resilience against (psychosocial) stress (Montgomery et al., 2006). Moreover, (extended) breastfeeding was believed to be associated with specific measures of infant attachment security (Gibbs et al., 2018) and upward social mobility (Sacker et al., 2013). Infants that are born small and/or experience suboptimal growth patterns after birth may benefit more from the positive effects of extended breastfeeding on growth (Kuchenbecker et al., 2015) and brain development (Anderson et al., 1999; Rao et al., 2002) compared to their normal birth weight peers.

Aspects that contribute to the improved growth and neurodevelopment observed in breastfed infants compared to formula-fed infants may include differences in the quality of dietary lipids between HM and IMF. The major energy component of HM and IMF comprises lipids, which do not only provide energy for growth but also supply building blocks and cofactors to allow growth and development of tissues and organs early in life. For example, long-chain poly-unsaturated fatty acids, such as docosahexaenoic acid and arachidonic acid in HM, are considered essential for brain development and function (Hadley et al., 2016; Weiser et al., 2016). In addition, milk lipids contribute to the absorption of fat-soluble vitamins, such as vitamin D, which is, in turn, essential for adequate absorption of calcium and skeletal growth and development (Laird et al., 2010). Whereas, the lipid content and composition of commercially available IMF resemble that of HM, many differences remain (Carlson et al., 2021). Another aspect of lipid quality is the supramolecular structure of lipids, which is quite different in HM compared to IMF. Mammalian milk lipid droplets are large, with an average mode diameter between 3 and 5 μm (Michalski et al., 2005), and consist of a triglyceride core, which is surrounded by a biological membrane (i.e., the so called milk fat globule membrane, MFGM) that is composed mainly of phospholipids (PL), cholesterol, and some other bioactive components (Lopez and Menard, 2011). Lipid globules in standard IMF, however, are small (a diameter of approximately 0.1–1 μm) and consist of a core of triglycerides with mostly proteins adhering to the surface but without a PL membrane. The size and surface properties of lipid globules are instrumental in absorption and digestion kinetics in the gastrointestinal tract after ingestion, thereby affecting appearance of lipids and post-prandial hormones in the circulation and their subsequent distribution and bioavailability to developing organs (Michalski et al., 2013; Bourlieu et al., 2015; Baumgartner et al., 2017). In addition, the absorption of fat soluble vitamins and cofactors may be altered by the complex supramolecular structure of lipids in milk (Bezelgues et al., 2009). Because lipids, vitamins, and hormones are important for structural and functional development of organs and tissues early in life, a different bioavailability that is caused by the more complex supramolecular structure in HM compared to IMF may explain some of the beneficial outcomes in growth and neurocognitive outcomes that are observed following (extended) breastfeeding.

In a previous study, young mice were exposed to an IMF containing enlarged lipid droplets coated by PL and other MFGM-derived ingredients (Nuturis^®^), thereby resembling the supramolecular lipid structure of mammalian milk more closely than standard IMF. The diet was introduced at 16 days of age, directly following normal lactation and was provided throughout adolescence until 42 days of age, thereby resulting in prolonged exposure to large lipid globules with a complex surface area as are present in mammalian milk. We demonstrated that this diet improved performance on specific cognitive tests in adulthood (Schipper et al., 2016). In that experiment, however, the mice were individually housed, which might have been important in the outcome of the experiment. Social isolation of rodents, in particular from a young age onwards, has been studied as an environmental challenge that may affect cognitive function and behavior (Fone and Porkess, 2008). The associated neurochemical and neuroanatomical aberrations may include differences in myelination patterns (Makinodan et al., 2012) and altered hypothalamic-pituitary-adrenal (HPA) activation and responsiveness (Biggio et al., 2014; Cacioppo et al., 2015). The underlying mechanisms for these alterations are not fully understood but are thought to include the lack of social experience-dependent neuronal activity during critical periods of brain development (Makinodan et al., 2012), as well as alterations in glucocorticoid feedback (Hawkley et al., 2012). Moreover, individual compared to social housing may alter growth and metabolic health outcomes in mice (Schipper et al., 2018). Compared to individual housing, social housing is considered a more naturally relevant environmental condition for mice, which allows normal growth and development.

Reproducibility and replicability are key factors in the reliability of study results. In the current study, we, therefore, evaluated the effects of exposure to the diet with a more mammalian milk-like supramolecular lipid structure, following normal lactation on cognitive function and associated parameters in individually as well as in socially housed mice. Moreover, we also evaluated effects of the diet on previously unexplored outcomes, including growth, bone length, and anxiety-like behavior. We hypothesized that individual compared to social housing of mice impairs growth and neurocognitive function, while prolonged exposure to dietary lipids with more mammalian milk like supramolecular lipid structure improves these outcomes, thereby preventing the detrimental effects of individual housing.

## Materials and Methods

### Animals and Study Design

Experimental animals (C57BL/6J) were bred in house; primiparous breeder dams and males were obtained from Charles River laboratories (Sulzfeld, Germany). All the animals were housed in a controlled environment (a 12/12-h light/dark cycle with lights on at 08:00, 21 ± 2°C) with *ad libitum* access to semi synthetic food (see below) and water, unless specified otherwise. All the animals were housed in Makrolon type III cages, containing Aspen wood shavings and a shelter. All experimental procedures applied in this study were conducted in accordance with principles of good laboratory animal care, following the EU directive 2010/63/EU for the protection of animals used for scientific research purposes and were approved by an independent ethics committee for animal experimentation (DEC-Consult, Soest, Netherlands). Dams were kept on the American Institute of Nutrition (AIN)-93G diet (Reeves et al., 1993) (Research Diet Services, Wijk bij Duurstede, Netherlands). After birth, at post-natal day (PN) 2, litters were randomized and culled to six pups (a male:female ratio, 4:2 or 3:3). From birth to PN16, mice offsprings were exclusively suckled. At PN16, the offsprings with their dams were randomly assigned to one of the two diets containing IMF and differing in supramolecular lipid structure: Control (CTR) and Active (ACT, Nuturis^®^) diet. The diets were provided in the home cage as soft dough, still allowing the offsprings also to suckle until weaning. PN16 is an age at which mouse pups are known to voluntarily start consuming solid foods next to milk, and therefore, allows for the dietary intervention to be started in a non-stressful way before weaning. After weaning (PN21), the dams and female pups were removed from the study and the male pups were marked by ear clipping, moved to a different room where they were either housed individually or housed with a littermate (two animals per cage; social) and remained on CTR or ACT diet until PN42 (i.e., 6 weeks of age). The cages were randomly placed in the racks in the same room, while no attempt was made to prevent visual, auditory or olfactory contact between the mice in neighboring cages and/or cages elsewhere in the room. The animals in the four possible diet-housing combinations (control diet and individual housing, CTR-IND; active diet and individual housing, ACT-IND; control diet and social housing; CTR-SOC; active diet and social housing, ACT-SOC) were further divided to evaluate the effects of diet during adolescence and while still on the diet (adolescence, 3–6 weeks of age), or to evaluate long-term effects of the diet, after being switched to a standard semi synthetic rodent diet (AIN-93M) at 6 weeks of age (adulthood, 6–18 weeks of age). For determination of a behavioral phenotype during adolescence, the animals were subjected to behavioral testing between PN35 to PN38. These animals were sacrificed at 6 weeks of age. For evaluation of behavioral phenotype in adults, the animals were subjected to behavioral testing from PN88 until PN92. These animals were sacrificed at 18 weeks of age. For all the animals, body weight was recorded on a weekly basis (average weights of litter with all six pups before weaning and individual body weights of the male offspring after weaning).

### Diets

All diets (Research Diet Services, Wijk bij Duurstede, Netherlands) were semisynthetic and had a micro- and macronutrient composition according to the American institute of Nutrition formulation (AIN)-93G purified diets for laboratory rodents (Reeves et al., 1993). The Active and Control diets were produced as previously reported (Schipper et al., 2016). In brief, both the Active and Control diets contained 28.3%, w/w IMF powder (Nuturis^®^ and regular IMF, respectively), complemented with protein and carbohydrates to match the AIN-93G composition. The lipid fraction was exclusively derived from the IMF powders. In contrast to regular IMF, lipid droplets in Nuturis^®^ are large and are coated by PL and other MFGM-derived ingredients (Gallier et al., 2015). Powdered rodent diets were mixed with water (28% w/w%) to form dough balls, which were freshly prepared daily and provided in the cage.

### Behavior Tests

Naïve animals were subjected to the behavior tests either during adolescence (i.e., from PN35 until PN38) or during adulthood (i.e., from PN88 until PN92). All behavior tests were conducted during the light phase by an observer blinded to the experimental conditions. To minimize potential variability induced by prior test history on the behavioral responses, the tests were always run in the same order for all the mice, from least to most stressful (i.e., open field, novel object recognition, three chamber social tests, and elevated plus maze, respectively). The animals were transported in their home cage to an adjacent test room and allowed to habituate at least 30 min before the test. For the individually housed animals, the order of testing within each group was randomized. Socially housed cage mates were always tested successively, although taken randomly from the cage.

#### Open Field

Locomotor activity and exploratory behavior were evaluated during adolescence (PN35) and adulthood (PN88) using the open field test as previously described (Schipper et al., 2016). The mice were placed in a square open field (50 cm × 50 cm) for a period of 5 min, and the total distance moved and the time spent in the center square were recorded and analyzed using Ethovision XT 11.5 (Noldus, Wageningen, Netherlands), using the nose, center, and tail point of the mouse for tracking.

#### Novel Object Recognition Test

The ability of the mice to recognize a change in a previously explored environment was studied at adolescent (PN35 and 36) and adult (PN88 and 89) age by subjecting the mice to the novel object recognition test. The test was conducted using the same arena as used for the open field with the benefit that the test environment was already familiar with the mice. The test included three 5-min trials: object familiarization, novel place, and novel object recognition, as described previously (Schipper et al., 2016), with minor adaptations to the protocol. In short, the mice were introduced to the center square of the arena in each trial, an hour after the open field test. During object familiarization, two identical ceramic objects were present in opposing corners. About 24 h later, during the novel place trial, the animals were re-introduced in the arena, and one object was moved from the original corner to a new corner, while the other object remained at its original position. An hour after the novel place trial, novel object recognition was performed, where the previously moved object was now replaced by a novel object of similar size and material, but different shape. The basic measure was the time (s) taken by the mice to explore the objects in the novel place and the novel object trial, using the nose, center, and tail point of the mouse for tracking (Ethovision^®^ XT software, Noldus, Wageningen, Netherlands). Performance was evaluated by calculating a recognition index: [*N/*(*N* + *F*)] where *N* is time spent exploring the object in the new location (the novel place trial) or the novel object (the novel object trial), and *F* is time spent exploring the object in the familiar location (the novel place trial) or the familiar object (the novel object trial). The recognition index was calculated for the first 2 min after introduction into the arena. A higher index reflects better memory performance.

#### Elevated Plus Maze

Anxiety-like behavior was tested at adolescent (PN38) and adult (PN92) age using an elevated plus maze test. The elevated plus maze was composed of two enclosed arms (50 cm in length and 10 cm in width) opposed perpendicularly by two open arms (same size), extending out from a central platform (10 cm × 10 cm), all elevated approximately 100 cm from the floor. The test is based on an approach avoidance conflict: the natural tendency of mice to explore novel environments and their preference to stay in dark, protected, unexposed places (i.e., the enclosed arms). Mice that are more anxious spend more time in the closed arms (Hogg, 1996). The mice were placed in the center of the maze, facing one of the open arms, and then allowed to explore the maze for 5 min. Exploration in the maze was recorded with a camera for later (manual) recording of time spent in each arm. Time spent in the arm was defined as the mouse being located with all four paws in the arm. The anxiety index can be calculated according to: [*O/*(*O* + *C*)], where *O* is time spent exploring the open arm and *C* is time spent exploring the closed arm, with a higher index indicating a lower anxiety score. The% of time spent in the open and closed arms was also calculated, taking also into account the potential influence of the time spent in the center of the maze.

#### Three-Chamber Social Test

The effect of the post-natal dietary intervention and social conditions on adult social interest and social recognition was assessed using a three-chamber social test at PN 90, with a protocol similar to that described by Kaidanovich-Beilin et al. (2011). The three-chamber test comprises a Plexiglas arena divided into three chambers, each 19 cm × 45 cm by elevated walls, with an open middle section, allowing access to each chamber. Inside the side chambers, a plexiglass container with bars is placed, which can hold a mouse with whom social interaction is possible through visual, auditory, and olfactory cues, but physical interaction is prevented. In total, there were three consecutive trials of 10 min (5 min between each session), and sessions were recorded for later analysis with Ethovision^®^ XT software (Noldus, Netherlands), using the nose, center, and tail points of the mouse for tracking. The mice were introduced to the center chamber in each trial. The first session served as a habituation trial to the arena with empty containers in the side chambers. In the second trial (the social interest trial), an unfamiliar adolescent (±6-week-old) male mouse (the stimulus mouse) was introduced in the container in one of the side chambers. The experimental mouse was allowed to explore and interact with the stimulus mouse. Time spent in the chamber containing the mouse vs. time spent in the empty chamber describes the social interest of the mouse. The chamber with the stimulus mouse was randomized for each mouse. In the third and final trial (the social recognition trial), a second and unfamiliar stimulus mouse (novel) was introduced in the container in the opposing chamber. The amount of the time spent in the chamber with the novel stimulus mouse compared to the chamber with the now familiar stimulus mouse is an indication of (the recognition of, or interest in) social novelty. In addition, a performance index was calculated for social interest and social novelty: [*N/*(*N* + *F*)] where *N* is the time spent in the chamber with the novel stimulus mouse, and *F* is the time spent exploring the empty chamber (social interest) or chamber with the familiar stimulus mouse (social recognition).

### Tissue Collection and Processing

Prior to sacrifice at 6 or 18 weeks of age, the mice were fasted for 6 h (during dark the phase). The mice were anaesthetized by isoflurane inhalation, which was followed by heart puncture and decapitation. Brains were dissected into different sub-regions, including the hypothalamus and prefrontal cortex and snap frozen in liquid nitrogen. Length and width of the right femur were measured using a digital micro-caliper. Blood was centrifuged at 2,600 g for 10 min. Plasma was subsequently separated and stored at −80°C until analysis of plasma hormones corticosterone (CORT), insulin-like growth factor-1 (IGF1), osteocalcin (OCN), according to the manufacturer’s instructions (EIA Corticosterone kit, Arbor Assays, Ann Arbor, MI, United States; an IGF1 ELISA kit, R&D Systems, Minneapolis, MN, United States; a Mouse OT/BGP ELISA kit, CUSABIO, Houston, TX, United States). Carcasses were dried till constant weight at 103°C [ISO 6496-198 (E)], and this was followed by fat extraction with petroleum ether (Boom BV, Meppel, Netherlands) in a soxhlet apparatus. All organ analyses were performed by a technician blinded to the experimental conditions.

### RNA Preparation and Quantification of Gene Expression

RNA was isolated from sub-regions of the brain using TRIzol (Invitrogen) and chloroform and extracted using an RNeasy kit (Qiagen). RNA concentrations were assessed using a NANODROP 2000 (Thermo Scientific). RNA was converted to cDNA using a GoScript Reverse Transcription (Promega) kit, containing oligo dTs. Quantitative real-time polymerase chain reaction (qPCR) was used to analyze the mRNA expression of genes; In hypothalamic tissue Oxytocin/Neurophysin I Prepropeptide (*OXT*; F-TGCAGCCCGGATGGCT, R-ATTCC CAGAAAGTGGGCTCAG), corticotropin-releasing hormone (*CRH*; F-GCCTGGGGAATCTCAACAGA, R-AGCAACACG CGGAAAAAGTT) and corticotropin-releasing hormone receptor 1 (*CRHR1*; F-GTGTCCGCTACAACACCA, R-GCACT TTGCTCTTCTTCTCTTC) mRNA were measured. In prefrontal cortex tissue mRNA expression of myelin-associated glycoprotein (*MAG*; F-CCTGGATCTGGAGGAGGT GA, R-TTCACTGTGGGGTTCCAAGG) and myelin basic protein (*MBP*; F-AGCACCACTCTTGAACACCC, R-CAG CCTCTCCTCGGTGAATC) gene expressions were measured. Using qPCR software analysis (qBase+), a thorough evaluation of reference genes in both tissues was done and Actin (*ACT*; F-CCAGATCATGTTTGAGACCTTCAA, R-ATGGGCACAGT GTGGGTGAC) and TATA-Box-binding protein (*TBP*; F-CCC CACAACTCTTCCATTCT, R-GCAGGAGTGATAGGGGTCAT) were selected as reference genes for the hypothalamus, and *ACT* and Calnexin (*CANX*; F-AGAGCTCAGCCTGGATCAATTC, R-TTGTAGTCCTCTCCACACTTATCTGG) for the prefrontal cortex. All the experimentally induced variation has been corrected according to the qBase + manual, and the obtained calibrated normalized relative quantity (QNRQ) values represent the differences in gene expression.

### Statistical Analysis

Statistical analyses were performed using SPSS 26 (IBM Software) and graphical design using GraphPad Prism (GraphPad Software, Inc.). Data from litters before weaning were analyzed using one-way ANOVA with the factor diet. All other data were analyzed by two-way ANOVA with factors diet and housing condition. Significant interaction effects were followed by one-way ANOVA, with Tukey’s HSD as *post-hoc* test for comparisons between groups. All data are presented as mean ± SEM and considered significantly different when *p* < 0.05. Statistical trends (0.05 < *p* < 0.1) were also reported. After weaning, each group included a total of 10-16 animals (Adolescence: CTR-IND, *n* = 14; ACT-IND, *n* = 14; CTR-SOC, *n* = 16; ACT-SOC, *n* = 14; adulthood: CTR-IND, *n* = 13; ACT-IND, *n* = 14; CTR-SOC, *n* = 10; ACT-SOC, *n* = 14). Data from one animal in the adult ACT-SOC group were excluded from all statistical analyses because of malocclusion; the animal was, however, kept in the study to avoid individual housing of its cage mate. A different animal in the Adult ACT-SOC group unexpectedly died before the end of the study, and data were not collected from this animal at PN126. Datapoints missing for other reasons are specified in the figure captions when applicable.

## Results

### Growth and Body Composition

#### Growth of Pups Until Weaning Age

**FIGURE 1:**
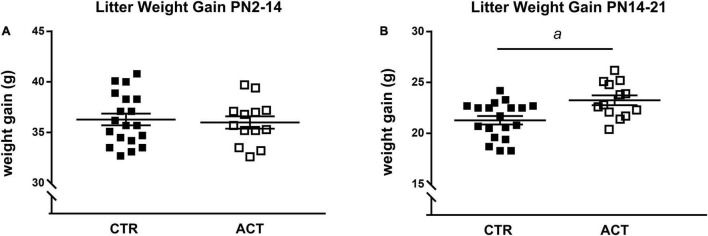
Effects of diet on body weight (gain) in litters until weaning. **(A)** Body weight gain of litters between PN2 and PN14 (prior to diet intervention). **(B)** Body weight gain of litters between PN14 and 21 (during diet intervention). Data are presented as mean ± SEM (CTR, *n* = 19; ACT, *n* = 13). *a* indicates a significant (main) effect of diet (*p* < 0.05).

All litters showed similar average weight gain during the first 2 weeks of life (Figure 1A). At PN16, the CTR and ACT diets were introduced. The litters exposed to ACT diet showed increased weight gain between P14 and 21 compared to that of litters exposed to CTR diet {main effect of diet [*F*_(1,36)_ = 1.122, *p* = 0.026], Figure 1B}.

#### Growth and Body Composition During Adolescence

**FIGURE 2:**
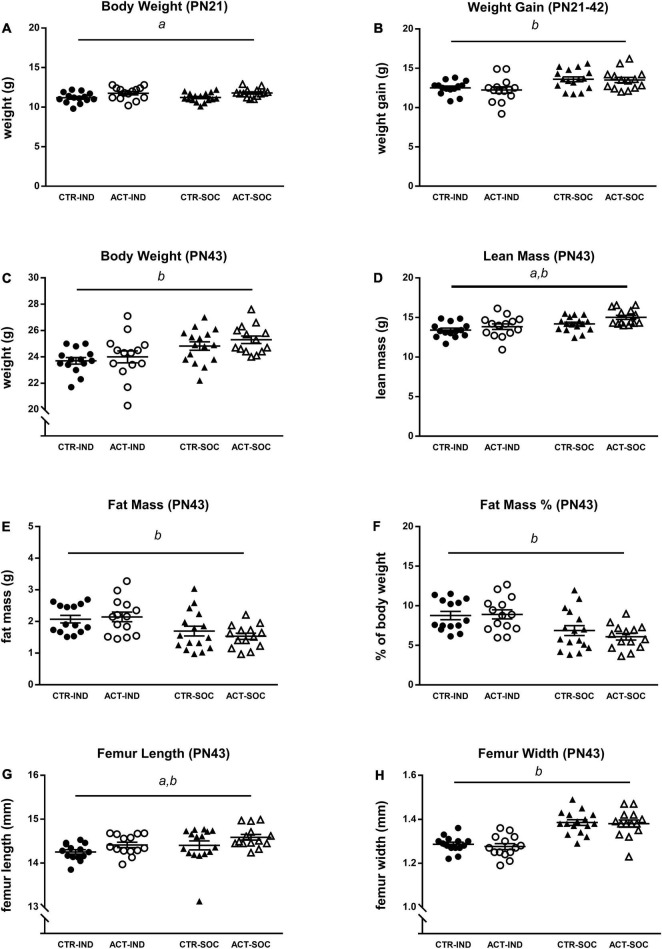
Effects of diet and housing on adolescent body weight and body composition. **(A)** Body weight at PN21 (baseline). **(B)** Adolescent body weight gain between PN21 and 42. **(C)** Body weight, **(D)** lean body mass (g), **(E)** fat mass (g), **(F)** body fat percentage, **(G)** femur length, and **(H)** femur width at PN43. Data are presented as mean ± SEM (CTR-IND, *n* = 14; ACT-IND, *n* = 14; CTR-SOC, *n* = 16; ACT-SOC, *n* = 14). *a* indicates a significant (main) effect of diet (*p* < 0.05) and *b* a significant (main) effect of housing condition (*p* < 0.05).

Effects of diet and housing conditions on growth and body composition were evaluated during adolescence while on CTR or ACT diets (between 3 and 6 weeks of age, PN21-PN42). Absolute body weight of animals at the baseline (PN21) was higher in ACT compared to CTR offsprings and not different depending on housing condition {main effect of diet, [*F*_(1,54)_ = 11.191, *p* = 0.001], Figure 2A}. Body weight gain during adolescence was reduced following individual housing [*F*_(1,54)_ = 15.752, *p* < 0.001] but was not significantly influenced by diet (Figure 2B). Absolute body weight at PN42 was significantly higher in socially housed animals versus individually housed animals {main effect of housing [*F*_(1,54)_ = 15.126, *p* < 0.001], Figure 2C}. At this age, individually housed animals had lower lean body mass {main effect of housing [*F*_(1,54)_ = 22.320, *p* < 0.001], Figure 2D}, increased fat mass {absolute (g), main effect of housing [*F*_(1,54)_ = 13.392, *p* = 0.001], Figure 2E}, and fat mass calculated as a percentage of body weight {*F*_(1,54)_ = 19.161, *p* < 0.001], Figure 2F}. Exposure to ACT diet increased lean mass {main effect of diet [*F*_(1,54)_ = 5.108, *p* = 0.028], Figure 2D}, irrespective of housing, while fat mass was not significantly affected by diet at this age. The length of the femur was measured as proxy or growth. Individually housed animals had reduced femur length {[*F*_(1,54)_ = 4.275, *p* = 0.043], Figure 2G} and femur width {main effect of diet [*F*_(1,54)_ = 55.509, *p* < 0.001, Figure 2H} compared to socially housed mice, whereas ACT diet increased femur length only {main effect of diet: [*F*_(1,54)_ = 4.834, *p* = 0.032], Figure 2G}, irrespective of housing condition.

#### Growth and Body Composition During Adulthood

**FIGURE 3:**
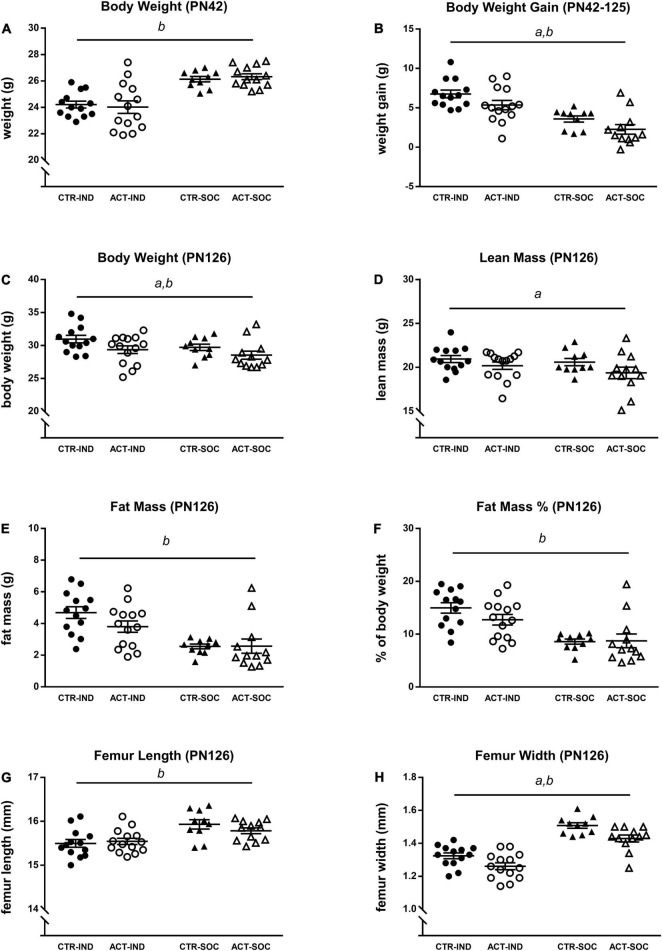
Effects of diet and housing on adult body weight and body composition. **(A)** Body weight at PN42 (baseline). **(B)** Adult body weight gain between PN42 and 126. **(C)** Body weight, **(D)** lean body mass (g), **(E)** fat mass (g), **(F)** body fat percentage, **(G)** femur length, and **(H)** femur width at PN126. Data are presented as mean ± SEM (CTR-IND, *n* = 13; ACT-IND, *n* = 14; CTR-SOC, *n* = 10; ACT-SOC, *n* = 12). *a* indicates a significant (main) effect of diet (*p* < 0.05), *b* a significant (main) effect of housing condition (*p* < 0.05) and a trending (main) effect of housing condition (0.05 < *p* < 0.10).

Effects of diet and housing conditions on growth and body composition were evaluated during adulthood, after previous exposure to CTR or ACT diet (between 6 and 18 weeks of age, PN42-PN126). Absolute body weight of animals at the baseline (PN42) was lower in individually compared to socially housed animals, but was not affected by diet {main effect of housing, [*F*_(1,46)_ = 29.348, *p* < 0.001], Figure 3A}. Body weight gain during adulthood was increased in individually housed mice compared to socially housed mice and by ACT diet {main effect housing [*F*_(1,46)_ = 30.325, *p* < 0.001], main effect diet [*F*_(1,46)_ = 5.241, *p* = 0.027], Figure 3B}. At PN126, absolute body weight was higher in individually compared to socially housed mice {main effect of housing [*F*_(1,46)_ = 4.231, *p* = 0.045], Figure 3C} and significantly decreased in animals previously exposed to ACT {main effect of diet [*F*_(1,46)_ = 7.007, *p* = 0.011], Figure 3C}. Dissection at this age revealed that lean body mass was decreased by previous ACT diet exposure {main effect of diet [*F*_(1,45)_ = 4.166, *p* = 0.047], Figure 3D} but not significantly by housing condition. Absolute fat mass (g) was, however, significantly higher in individually housed animals {main effect housing [*F*_(1,45)_ = 21.024, *p* < 0.001], Figure 3E}, resulting in a higher% of body fat at PN126 {main effect of housing [*F*_(1,45)_ = 25.453, *p* < 0.001], Figure 3F}. Changes in body composition were associated with a reduced femur length {main effect housing [*F*_(1,45)_ = 16.695, *p* < 0.001], Figure 3G} and femur width {main effect housing [*F*_(1,45)_ = 79.025, *p* < 0.001], Figure 3G} as a result of individual housing, whereas previous ACT exposure decreased femur width only {main effect of diet: [*F*_(1,45)_ = 12.81, *p* = 0.001], Figure 3H}, irrespective of housing condition.

### Behavior Tests

#### Open Field

**FIGURE 4:**
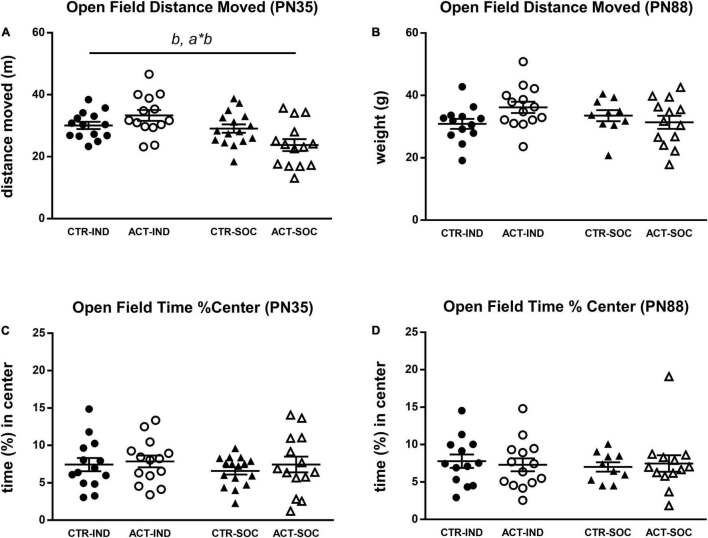
Effects of diet and housing on parameters in the open field test during adolescence (PN35) and adulthood (PN88). **(A)** Distance (m) moved in adolescent animals. **(B)** Distance (m) moved in adult animals. **(C)** Time (%) spent in the center during adolescence. **(D)** Time (%) spent in the center during adulthood. Data are presented as mean ± SEM (adolescence: CTR-IND, *n* = 14; ACT-IND, *n* = 14; CTR-SOC, *n* = 16; ACT-SOC, *n* = 14; adulthood: CTR-IND, *n* = 13; ACT-IND, *n* = 14; CTR-SOC, *n* = 10; ACT-SOC, *n* = 13). *b* indicates a significant (main) effect of housing (*p* < 0.05); a*b indicates a significant interaction effect diet*housing (*p* < 0.05).

Effects of diet and housing conditions on locomotor activity and exploratory behavior of mice were evaluated using the Open Field test. In adolescent animals (PN35), the total distance moved (cm) traveled in the OF was increased by individual housing {main effect of housing, [*F*_(1,54)_ = 11.515, *p* = 0.001], Figure 4A}. For this parameter, there was a significant interaction between housing and diet [*F*_(1,54)_ = 7.473, *p* = 0.008], which was driven by higher locomotor activity by ACT exposure in individually housed animals (*p* < 0.001). Locomotor behavior during adulthood was not influenced by diet or housing (Figure 4C). The time spent in the center during adolescence and adulthood was also not influenced by diet or housing (Figures 4B,D).

#### Novel Object Recognition Test

**FIGURE 5:**
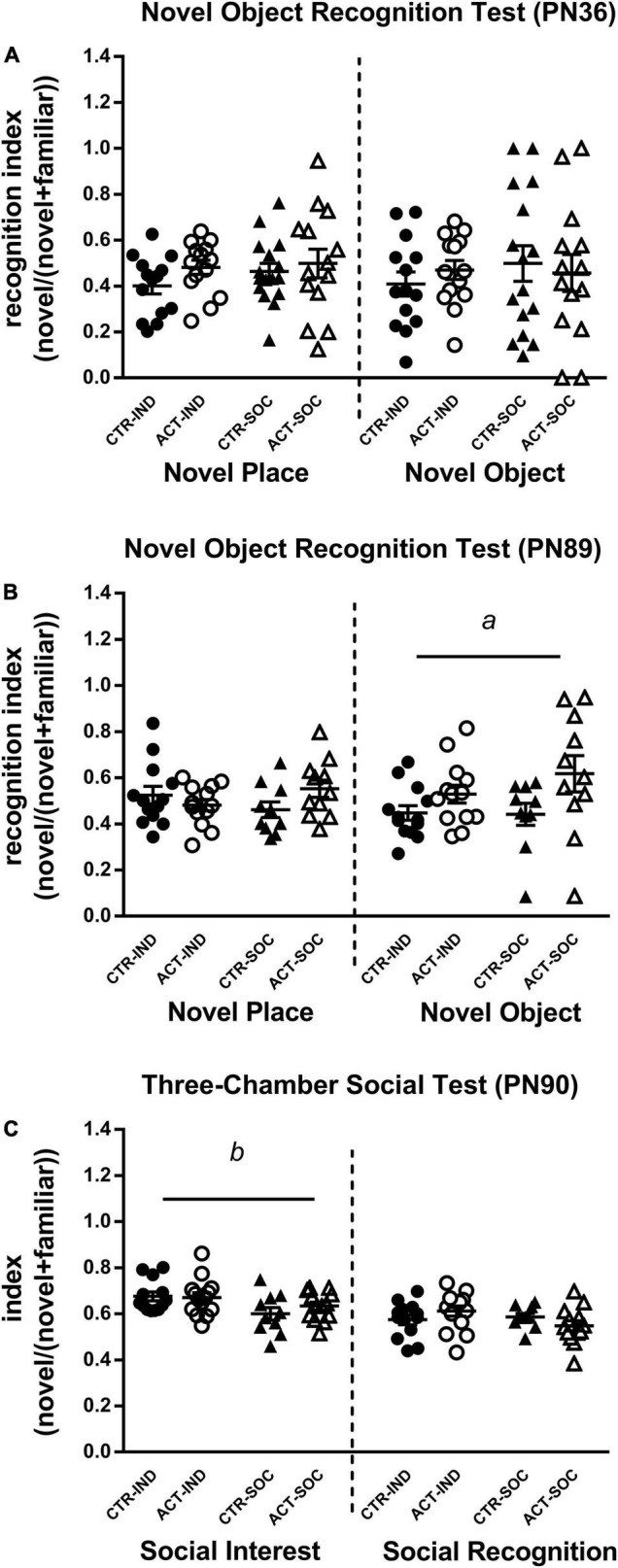
Effects of diet and housing on parameters in the novel object recognition test during adolescence (PN36) and adulthood (PN89) and the three-chamber social test during adulthood (PN90). **(A)** A novel place and novel object recognition index during adolescence. **(B)** A novel place and novel object recognition index during adulthood. **(C)** Social interest and social recognition during adulthood. Data are presented as mean ± SEM (adolescence: CTR-IND, *n* = 14; ACT-IND, *n* = 14; CTR-SOC, *n* = 16; ACT-SOC, *n* = 14; adulthood: CTR-IND, *n* = 13; ACT-IND, *n* = 13–14; CTR-SOC, *n* = 10; ACT-SOC, *n* = 11-13). Three data points in panel **(B)** and one data point in panel **(C)** are missing because the technical issues data were not properly recorded during these sessions. *a* indicates a significant (main) effect of diet (*p* < 0.05) and *b* a significant (main) effect of housing condition (*p* < 0.05).

The Novel Object Recognition test was used to study effects of diet and housing conditions on cognitive function. Neither diet nor housing significantly affected the discrimination index during the object location and novel object trial in adolescent (PN36) animals (Figure 5A). In adulthood (PN89), however, the recognition index during novel object trial was significantly increased by early life ACT diet exposure irrespective of housing condition {main effect of diet [*F*_(1,43)_ = 6.464, *p* = 0.015], Figure 5B}.

#### Elevated Plus Maze

**FIGURE 6:**
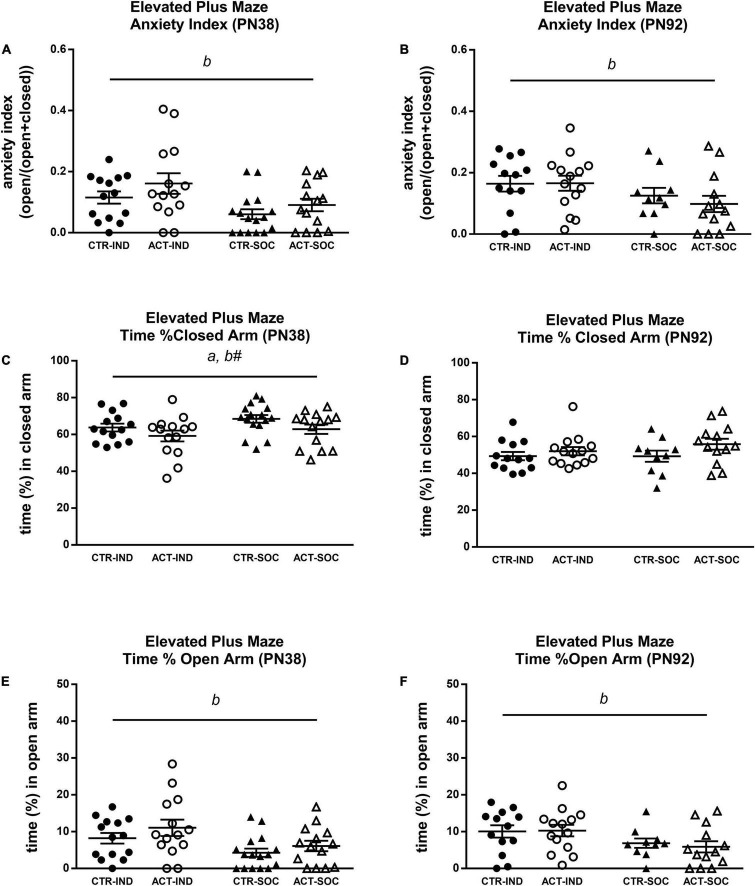
Effects of diet and housing on parameters in the elevated plus maze test during adolescence (PN38) and adulthood (PN92). **(A)** Anxiety index in adolescent animals, a higher index indicates lower anxiety-like behavior. **(B)** Anxiety index in adult animals. **(C)** Time (%) spent in the closed arm during adolescence. **(D)** Time (%) spent in the closed arm during adulthood. **(E)** Time (%) spent in the open arm during adolescence. **(F)** Time (%) spent in the open arm during adulthood. Data are presented as mean ± SEM (adolescence: CTR-IND, *n* = 14; ACT-IND, *n* = 14; CTR-SOC, *n* = 16; ACT-SOC, *n* = 14; adulthood: CTR-IND, *n* = 13; ACT-IND, *n* = 14; CTR-SOC, *n* = 10; ACT-SOC, *n* = 13). *a* indicates a significant (main) effect of diet (*p* < 0.05), *b* a significant (main) effect of housing condition (*p* < 0.05), and *b#* a trending (main) effect of housing condition (0.05 < *p* < 0.10).

Effects of diet and housing conditions on anxiety-like behavior of mice were evaluated using the Elevated Plus Maze test. At PN38, individual housing increased the anxiety index compared to social housing {main effect of housing [*F*_(1,54)_ = 7.284, *p* = 0.009], Figure 6A}, reduced the time spent in the closed arm, albeit not significantly {trending effect of housing [*F*_(1,54)_ = 2.924, *p* = 0.093], Figure 6C}, and the increased time spent in the open arm of the elevated plus maze {main effect of housing [*F*_(1,54)_ = 8.016, *p* = 0.007], Figure 6E}. The animals exposed to the ACT diet spent less time in the closed arms compared to the animals exposed to CTR {main effect of diet [*F*_(1,54)_ = 4.349, *p* = 0.042], Figure 6C}. The higher anxiety index due to individual housing was also observed in adulthood (PN92) {main effect of housing [*F*_(1,46)_ = 4.200, *p* = 0.046], Figure 6B} and the individually housed animals spent less time in the open arm {main effect of housing {*F*_(1,46)_ = 5.946, *p* = 0.019], Figure 6F}, but there were no effects of early life diet exposure on any of the parameters in the elevated plus maze (Figure 6D).

#### Three-Chamber Social Test

The mice were subjected to the Three-Chamber Social test to study effects of diet and housing condition on adult social interest and social recognition. During the social interest trial in adulthood (PN 90), all the mice showed a strong preference for the chamber containing the stimulus mouse compared to the empty chamber, indicating normal social interest, social motivation, and affiliation. The social interest index was increased by individual housing but not by diet {main effect of housing, index [*F*_(1,46)_ = 8.838, *p* = 0.005], Figure 5C}. During the social recognition trial, all the mice showed a strong preference for the chamber with the novel stimulus mouse, which may indicate a preference for novelty and intact social memory, but recognition index was not affected by diet or housing. There were no signs of aggressive behaviors during direct interaction with the stimulus mouse (e.g., bar biting, tail rattling).

### Plasma Hormones

**FIGURE 7:**
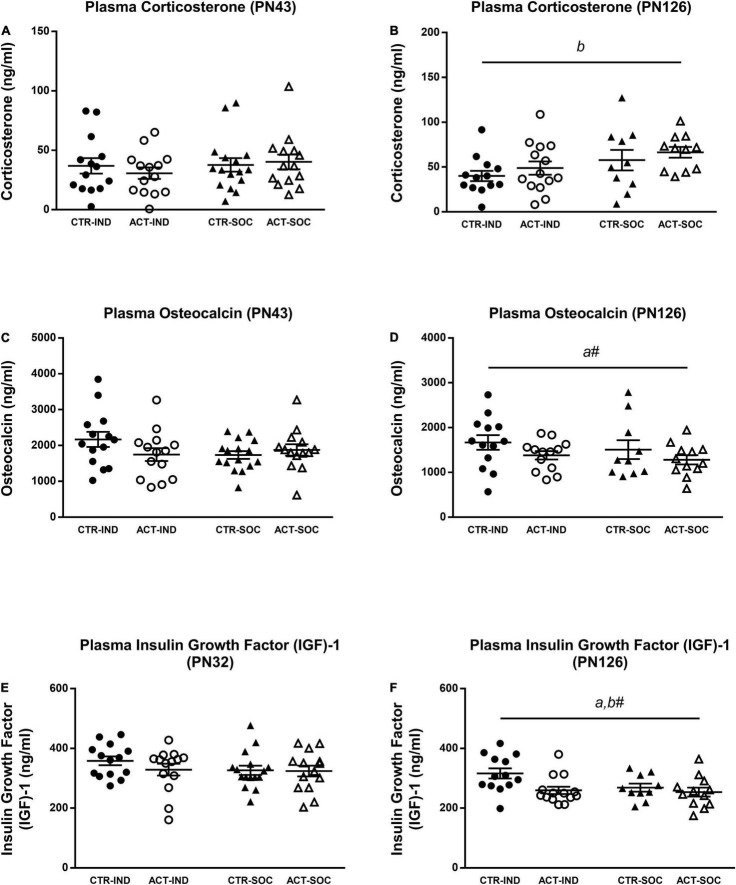
Effects of diet and housing on parameters on plasma hormones at adolescent (PN43) and adult age (PN126). **(A)** Corticosterone at adolescent age. **(B)** Corticosterone at adult age. **(C)** Insulin growth factor (IGF)-1 at adolescent age. **(D)** IGF-1 at adult age. **(E)** Osteocalcin at adolescent age. **(F)** Osteocalcin at adult age. Data are presented as mean ± SEM (adolescence: CTR-IND, *n* = 14; ACT-IND, *n* = 14; CTR-SOC, *n* = 16; ACT-SOC, *n* = 14; adulthood: CTR-IND, *n* = 13; ACT-IND, *n* = 13–14; CTR-SOC, *n* = 10; ACT-SOC, *n* = 11–12). Two data points are missing in panels **(B,D)**, respectively, due to insufficient amount of plasma. *a* indicates a significant (main) effect of diet (*p* < 0.05), *a#* a trending (main) effect of diet (0.05 < *p* < 0.10), and *b#* a significant (main) effect of housing condition (*p* < 0.05), a trending (main) effect of housing condition (0.05 < *p* < 0.10).

Plasma corticosterone levels in the adolescent mice were not influenced by diet or housing (Figure 7A); however, in the adult mice, plasma corticosterone was increased in socially housed animals {main effect of housing, [*F*_(1,44)_ = 5.192, *p* = 0.02], Figure 7B}. Plasma insulin growth factor (IGF)-1 did not differ in the adolescent animals; however, at adulthood, it was significantly decreased in the animals previously exposed to ACT diet {main effect of diet, [*F*_(1,45)_ = 5.846, *p* = 0.02], Figure 7D}. Adult IGF-1 plasma levels appeared to be lower (trend) in socially housed animals compared to individually housed animals {main effect of housing, [*F*_(1,45)_ = 3.212, *p* = 0.08], Figure 7D}. Plasma osteocalcin (OCN) was not affected by diet or housing during adolescence (Figure 7C), and, while in adulthood, OCN appeared lower in the animals that were fed ACT during early life; this was not significant {main effect of diet, [*F*_(1,46)_ = 3.142, *p* = 0.08], Figure 7D}. Plasma insulin growth factor (IGF)-1 did not differ in the adolescent animals (Figure 7E); however, at adulthood, it was significantly decreased in the animals previously exposed to ACT diet {main effect of diet, [F_(1,45)_ = 5.846, *p* = 0.02], Figure 7F}. Adult IGF-1 plasma levels appeared to be lower (trend) in socially housed animals compared to individually housed animals {main effect of housing, [F_(1,45)_ = 3.212, *p* = 0.08], Figure 7F}.

### Gene Expression in Hypothalamus and Prefrontal Cortex

**FIGURE 8:**
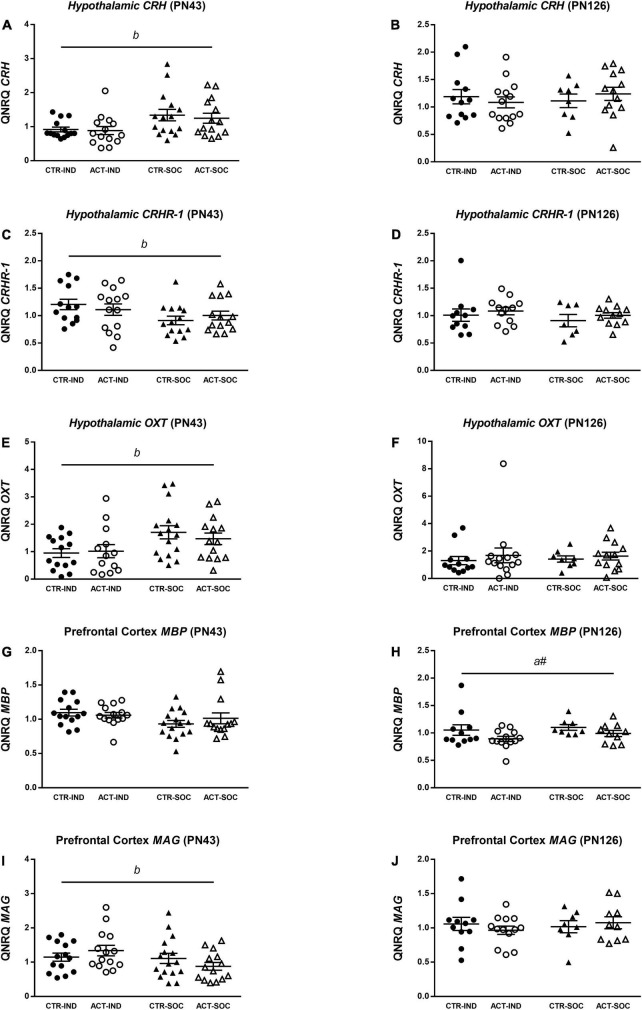
Effects of diet and housing on parameters on mRNA expression at adolescent (PN43) and adult age (PN126). **(A)** Hypothalamic *CRH* at adolescent age. **(B)** Hypothalamic *CRH* at adult age. **(C)** Hypothalamic *CRHR-1* at adolescent age. **(D)** Hypothalamic *CRHR-1* at adult age. **(E)** Hypothalamic *OXT* at adolescent age. **(F)** Hypothalamic *OXT* at adult age. **(G)** Prefrontal cortex *MBP* at adolescent age. **(H)** Prefrontal cortex *MBP* at adult age. **(I)** Prefrontal cortex *MAG* at adolescent age. **(J)** Prefrontal cortex *MAG* at adult age. Data are presented as mean ± SEM (adolescence: CTR-IND, *n* = 13–14; ACT-IND, *n* = 13–14; CTR-SOC, *n* = 14–16; ACT-SOC, *n* = 14; adulthood: CTR-IND, *n* = 11–12; ACT-IND, *n* = 12–14; CTR-SOC, *n* = 7–8; ACT-SOC, *n* = 10–13). One data point is missing in panels **(A,E)**, three data points are missing in panels **(B,C,F)**, seven data points are missing in panels **(H,J)**, and eight data points are missing in panel **(D)** due to technical issues. *b* indicates a significant (main) effect of housing condition (*p* < 0.05) and *a#* a trending (main) effect of diet (0.05 < *p* < 0.10).

In the adolescent individually housed animals, hypothalamic mRNA expression of corticotropin-releasing hormone (*CRH*) was lower {main effect of housing, [*F*_(1,54)_ = 8.698, *p* = 0.005], Figure 8A}, while corticotropin-releasing factor receptor 1 (*CRHR1*) was increased {main effect of housing, [*F*_(1,54)_ = 4.966, *p* = 0.030], Figure 8C} compared to socially housed animals. Hypothalamic Oxytocin (*OXT*) mRNA expression was reduced by individual housing at adolescence {main effect of housing, [*F*_(1,54)_ = 7.751, *p* = 0.007], Figure 8E}. There were no effects of diet on hypothalamic *CRH*, *CRHR1* or *OXT* expression at adulthood (Figures 8B,D,F). In adolescent animals, prefrontal cortex mRNA expression of myelin-basic protein (*MBP*) was not affected by housing or diet (Figure 8G), while myelin-associated glycoprotein (*MAG*) mRNA expression was higher in the adolescent individually housed animals compared to the socially housed animals {main effect of housing, [*F*_(1,54)_ = 5.200, *p* = 0.027], Figure 8I} At adulthood, *MBP* mRNA expression appeared to be lower in animals on an ACT diet {trending effect of diet [*F*_(1,38)_ = 4.038, *p* = 0.052], Figure 8H}; *MAG*, however, did not differ between groups (Figure 8J).

## Discussion

In the current study, the individually and socially housed male mice were exposed during adolescence to a diet with large, PL-coated lipid droplets, mimicking the supramolecular structure of lipids in mammalian milk. Exposure to this diet started at post-natal Day (PN) 16, which is directly after normal lactation, and, in this model, the mice, therefore, experience prolonged exposure to large lipid globules with a complex surface area as are present in mammalian milk. The results show that this diet increased femur length and lean body mass in the mice at PN43, without affecting body fat content. In adulthood, however, the growth rates were reversed, with reductions in body weight and lean mass in the animals exposed to ACT early in life. Femur length as a proxy for body length, however, was not affected by ACT in adulthood. These effects of early life dietary exposure to ACT coincided with lower anxiety-like behavior levels during/in adolescence, and improved novel object recognition in adulthood. While the social housing condition influenced growth and affective functioning, there does not seem to be a relevant interaction effect between housing condition and ACT exposure on metabolic and behavioral phenotypes. Taken together, these results suggest that ACT diet may modify the physical and behavioral phenotypes irrespective of (social) housing environment.

The effect of the ACT diet in the present study on the increase of lean body mass during adolescence confirms the results of a previous study in mice (Oosting et al., 2012). Interestingly, we observed in the current study, that body weight was increased at weaning on PN21 due to the early ACT exposure. The ACT diet was introduced to the litters at PN16, and PN21 was the first time point at which body weight was recorded thereafter. The exact age at which ACT and CTR animals first start to deviate in body weight, therefore, remains to be determined in future experiments. In humans, breastfed infants show an initially faster growth rate during the first few months of life compared to formula-fed infants (Butte et al., 2000; Kramer et al., 2002). Whereas, breastfed infants have higher body fat and lower lean mass than formula-fed infants at least up to 6 months of age (Gale et al., 2012), this switches to a reduced adiposity build-up and increased lean mass during childhood and adolescence (Gillman et al., 2001; Arenz et al., 2004; Gale et al., 2012). Moreover, breastfeeding (duration) may be associated with improved bone health in childhood and adolescence (Muniz et al., 2015). The more complex supramolecular structure of lipids in HM compared to that in formula may be one of the factors that contribute to the differences in the quality of growth and (skeletal) development observed between breastfed and formula-fed infants up to adolescence. One possibility is that both lipid droplet size and complex surface properties of dietary lipid globules influence nutrient absorption and digestion kinetics, which influences the bioavailability of nutrients to developing organs (Michalski et al., 2013; Bourlieu et al., 2015; Baumgartner et al., 2017). This may also include a different absorption of fat soluble vitamins and related cofactors, such as vitamin D and calcium that, together, stimulate bone growth (Laird et al., 2010). While studies about the effects of breastfeeding on bone characteristics in adulthood remain inconclusive (Pearce et al., 2010; Pirila et al., 2011), breastfeeding (duration) is believed to have a moderate but consistent protective effect against obesity later in life (Oddy et al., 2014). In line with this, previous studies in (socially housed) mice repeatedly showed that early-life dietary exposure to ACT resulted in reduced body fat accumulation and body weight (gain) in adulthood when challenged with a high-fat diet, e.g., Oosting et al. (2014) and Baars et al. (2016). While adult body weight (gain) was also lower due to ACT in the current study, this effect did not appear to be caused by reduced body fat accumulation. In contrast to the previous studies evaluating effects of ACT diet on (adult) metabolic health outcomes, the animals in the current study were kept on regular semi-synthetic rodent chow in adulthood rather than a high-fat-diet challenge. While individual housing is also known to result in higher body fat accumulation in mice (Schipper et al., 2018), we did not see a permissive nor modulatory effect of the housing condition on the effect of ACT on adult body weight (gain) and body composition. The reduced body weight at PN126 in the animals previously exposed to ACT was moderate and was observed together with reduced lean mass and subtle reductions in femur width, but not femur length. Body weight and bone width, but not length, are correlated to support mechanical loading (Molgaard et al., 1998). Aligned with these effects, we observed that early-life exposure to ACT reduced plasma IGF-1 levels in adulthood, but not during adolescence. While IGF-1 acts as a regulator of post-natal (skeletal) growth and organ maturation during early-life phases (Racine and Serrat, 2020), it is also known to be involved in a variety of metabolic functions in adulthood (Kineman et al., 2018). It may be speculated that the effects of ACT are mediated *via* lowering of IGF-1 signaling; however, whether or not reductions in IGF-1 signaling are beneficial for sustainable health is currently under debate (Vitale et al., 2019). Previous studies showed that, at adulthood, dampening free IGF-1 levels (by knockout of *PAPP-A*, preventing the cleavage of IGF-1 BP at binding sites) improves a healthy life span in mice associated with improved cardiovascular health (Bale et al., 2017).

In the current study, cognitive function in adulthood was improved in the mice that were exposed to ACT early in life. These effects were observed in both the individually and socially housed mice and confirm previous findings in the individually housed mice (Schipper et al., 2016). Additionally, the ACT diet appeared to reduce anxiety-like behavior during adolescence. In analogy to humans, breastfeeding (duration) is suggested to not only be associated with improved cognitive function (Belfort et al., 2013; Horta et al., 2015; Leventakou et al., 2015) but also with reduced anxiety-related problems (Montgomery et al., 2006; Hayatbakhsh et al., 2012; Huang et al., 2019). The results from the current study suggest that the extended exposure to the supramolecular structure of lipid droplets in HM could, at least, in part, contribute to these effects. The exact mechanism by which the dietary lipid structure leads to alterations in cognitive function and anxiety is, however, not yet fully understood. The effects of diet were observed in behavior tests that involve novelty exposure, and it was previously hypothesized that the mice exposed to ACT diet may experience higher novelty-induced arousal than the mice exposed to CTR, affecting cognitive function (Schipper et al., 2016). Neural correlates of novelty-induced arousal include activity of the locus coeruleus noradrenergic system that projects to the brain area like the prefrontal cortex and hippocampus, thereby facilitating learning and memory performance (Berridge, 2008; Sara and Bouret, 2012; Zerbi et al., 2019). Following this notion, higher baseline arousal by ACT diet may be mediated by different post-prandial release of satiety hormones after ingestion of ACT compared to that of control diet. A study from 2017 in adult men, indeed, showed that an experimental IMF that mimics the supramolecular structure of lipid droplets in HM elicited prolonged release of the small intestine-derived satiety hormone cholecystokinin (Baumgartner et al., 2017). Cholecystokinin has been shown to simulate activity in the locus coeruleus (Monnikes et al., 1997; Hwang et al., 2013) and may thereby augment arousal and facilitate learning and memory processes (Gulpinar and Yegen, 2004). Exposure to ACT diet during early life may have long-term influences on novelty-induced arousal or stress response as the development of locus coeruleus neurons and their projections to target areas continues during post-natal life in rodents (McLean and Shipley, 1991; Saito et al., 1996; Pinos et al., 2001), and noradrenalin acts as an important neurotropic factor during this developmental period (Marshall et al., 1991; Sullivan et al., 1991; Siciliano et al., 1999). Future studies may focus on the effects of ACT diet on the structure or function of the locus coeruleus and its target areas to confirm this hypothesis. In addition, evaluation of the effects diet on behavioral performance during the dark phase in future studies would be interesting. In the current study, the behavioral tests took place during the light phase, which is the active phase for mice. The relationship between arousal and cognitive processes is known to be influenced by circadian rhythms (Fisk et al., 2018). In any event, the effects of diet on behavior performance did not pertain to alteration in the baseline levels of plasma glucocorticoids and hypothalamic expression of *CRH* and its receptor *CRHR-1*. These, however, were found to be elevated by the social housing condition, which may presumably be linked to a more socially demanding housing environment. Early-life dietary lipid structure did not appear to result in long-term differences in social behavior in the current study as adult social interest as well as recognition in the three-chamber social test remained unaffected by diet. Oxytocin is a hormone that is involved in the regulation of social and emotional behavior, and its hypothalamic expression may depend on social stimuli (Resendez et al., 2020). While reduced hypothalamic *OXT* expression was observed together with alterations in social behavior and anxiety-like behavior in individually compared to socially housed animals, diet did not affect *OXT* expression. In humans, the positive association between breastfeeding and later-in-life social status maybe accounted for by improved cognitive development and reduced anxiety (Sacker et al., 2013).

Improved cognitive function and reduced anxiety-like behavior as seen in the ACT-fed mice may also be related to the increased lean mass and skeletal development in these animals at early age. Skeletal development and homeostasis may contribute to brain development, neuronal structure, and behavioral function (Chen et al., 2021). For instance, bone tissue secretes the hormone osteocalcin (OCN) based on the maturity of osteoblasts and the availability of vitamin K (Gundberg et al., 2012; Neve et al., 2013), and OCN has been shown to promote synthesis of several neurotransmitters, including serotonin, dopamine, and noradrenalin during post-natal life, affecting cognitive and behavioral phenotypes (Oury et al., 2013; Berggren et al., 2022). In the current study, circulating OCN levels were not significantly affected by diet at adolescent age (post-natal day 43), which is, however, a time point that is beyond bone development at its highest rate (Sanger et al., 2011). OCN secretion may be more sensitive to dietary factors at earlier stages of development. Maturation of the prefrontal cortex, an important area involved in the regulation of cognitive function, continues until late adolescence in mice, and its development is sensitive to environmental factors, including nutrition (Labouesse et al., 2018) and social interaction (Makinodan et al., 2012). While prefrontal cortex hypomyelination was associated with post-weaning social isolation (Makinodan et al., 2012), cognitive impairments that were observed in offsprings after perinatal high-fat diet exposure were associated with reduced expression of *MBP* in the hippocampus, but not the prefrontal cortex (Bordeleau et al., 2021), which suggests that the mechanisms by which these environmental factors may target the prefrontal cortex may be different. In the current study, diet did not affect prefrontal cortex *MBP* and *MAG* mRNA expression at adolescent age. The improvements cognitive performance by ACT were, however, observed together with a trend for reduced *MBP* mRNA expression at adult age. This effect remains unexplained, underlying a mechanism by which dietary lipid structure early in life may influence myelination at later age warrants further investigation. We did not observe reduced *MBP* and *MAG* mRNA expression in the prefrontal cortex as a result of post-weaning individual housing that had been previously reported (Makinodan et al., 2012), which could potentially be related to differences between studies in experimental design and or methods used, including the number of animals in the socially housed group. Other differences in experimental design that may impact experimental outcomes in relation to (social) stress-induced impairments in rodents include diet type (Goto et al., 2016) and handling intensity (Song et al., 2021).

Important for consideration of our findings is that the ACT diet used in the current study contained PL sourced from bovine MFGM, not present in the control diet. Several studies suggest that supplementation with MFGM-derived fragments may have a positive impact on cognitive outcomes of formula-fed infants, bringing them closer to the level of breastfed infants (Gurnida et al., 2012; Timby et al., 2014; Li et al., 2019). In experimental studies, the relevance of dietary supply for structural and functional brain development has been confirmed for some of the components present in MFGM, such as sphingomyelin (Schneider et al., 2019) and sialic acid (Wang, 2012). In addition, some of these components were reported to contribute to skeletal health (Khavandgar and Murshed, 2015; van Karnebeek et al., 2016). Although a role for the presence of the PL as an ingredient in the ACT diet cannot be excluded in the current study, it is postulated that the lipid structure closer to mammalian milk, e.g., the large lipid droplets coated by PL (Gallier et al., 2015) contributed, at least partially, to the observed improvement in growth, skeletal development, and behavioral outcomes. We previously showed that the unique combination of large and MFGM-PL-coated lipid droplets, but not the individual components, generated metabolic health benefits (Baars et al., 2016). In addition, the behavioral benefits as reported here were achieved with a relatively low dose of MFGM-derived PL in contrast to the dose previously reported to be effective when added as an ingredient to rodent diet (Vickers et al., 2009). The potential role of the microbiota modulating the effects of ACT diet on neurocognitive changes warrants further investigation. Gut microbiota is increasingly being recognized as an important regulator for brain development (Carlson et al., 2018). During infancy, the microbiome rapidly changes and is sensitive to infant diet (Stewart et al., 2018). Indeed, recent clinical studies have shown that dietary supplementation with MFGM can modulate infant gut microbiota (He et al., 2019; Chichlowski et al., 2021). It remains to be determined in future studies whether also the supramolecular structure of dietary lipids can influence gut microbiota.

The beneficial effects of breastfeeding (duration) on infant growth, metabolic health, and brain development and function have been a subject of debate, since other environmental conditions, such as parental health and behavior, as well as social economic factors, may ameliorate the effects (Walfisch et al., 2013; Durmus et al., 2014). Especially infants facing challenges, such as suboptimal growth and preterm birth, may be more susceptible to the protective effect of breastfeeding, while such positive effects are more difficult to show in healthy term infants (Anderson et al., 1999; Rao et al., 2002; Kuchenbecker et al., 2015). Individual housing impaired early life growth, and, in line with this, we anticipated potentially stronger effects of the ACT diet on outcomes in the individual housed mice compared to the social housed mice in the current study. Surprisingly, there were no significant diet* housing interactions in the current study, and the ACT effects were, in fact, similar on the outcomes evaluated irrespective of housing conditions. The results of the current study, therefore, do not directly support the idea that protective effects of (prolonged) breastfeeding may be confounded by other environmental conditions. In our previous study, we showed that early-life exposure to ACT diet positively affects adult cognitive function in the individually housed mice (Schipper et al., 2016). In the current study, we reproduced these findings and replicated the outcomes in socially housed animals. Clinical trials evaluating the effects of an IMF with large, PL-coated lipid droplets on infant growth and neurocognitive function are currently ongoing (Shek et al., 2021).

In summary, we found that exposure to a diet containing lipids with a supramolecular structure mimicking the lipid structure present in mammalian milk following normal lactation improved adolescent growth and (skeletal) development, while reducing adolescent anxiety and improving adult cognitive function. These effects were observed in both the socially and individually housed male mice, the latter reflective of a challenged early-life-growth condition. We suggest that the supramolecular structure of dietary lipids in HM may contribute to improvements in growth, skeletal development, body composition, and neuronal development as observed in breastfed infants. Based on these results, we propose that the supramolecular structure of lipids may provide an interesting aspect of dietary lipid quality in infant formula and follow-on formula that could contribute to healthy outcomes for both healthy infants, as well as infants facing growth challenges.

## Data Availability Statement

The original contributions presented in the study are included in the article/supplementary material, further inquiries can be directed to the corresponding author.

## Ethics Statement

The animal study was reviewed and approved by an independent ethics committee for animal experimentation (DEC-Consult, Soest, Netherlands).

## Author Contributions

GD, EB, and LS contributed to conception and design of the study. SH, GK, and LS performed the experiments and analyzed the data. SH and LS wrote the initial draft of the manuscript. All authors contributed to interpretation of results, manuscript revision, and read and approved the submitted version of the manuscript.

## Conflict of Interest

This study was funded by Danone Nutricia Research (DNR). LS and EB were employed by DNR at the time that this study was conducted. The remaining authors declare that the research was conducted in the absence of any other commercial or financial relationships that could be construed as a potential conflict of interest.

## Publisher’s Note

All claims expressed in this article are solely those of the authors and do not necessarily represent those of their affiliated organizations, or those of the publisher, the editors and the reviewers. Any product that may be evaluated in this article, or claim that may be made by its manufacturer, is not guaranteed or endorsed by the publisher.
